# Assessing the suitability and dynamics of three medicinal *Sambucus* species in China under current and future climate scenarios

**DOI:** 10.3389/fpls.2023.1194444

**Published:** 2023-10-19

**Authors:** Weixue Luo, Shunxin Han, Ting Yu, Peng Wang, Yuxuan Ma, Maji Wan, Jinchun Liu, Zongfeng Li, Jianping Tao

**Affiliations:** ^1^Key Laboratory of Eco-environments in Three Gorges Reservoir Region (Ministry of Education), Chongqing Key Laboratory of Plant Ecology and Resources Research in Three Gorges Reservoir Region, School of Life Sciences, Southwest University, Chongqing, China; ^2^Chongqing Jinfo Mountain Karst Ecosystem National Observation and Research Station, Southwest University, Chongqing, China

**Keywords:** climate change, *Sambucus*, medicinal plants, habitat distribution, random forest, species distribution model

## Abstract

Climate change exerts profound influences on the ecological environments on a global scale, leading to habitat destruction and altering distribution patterns for numerous plant species. Traditional Chinese medicinal plants, such as those belonging to the *Sambucus* genus, have been extensively utilized for several centuries to treat fractures, rheumatism, and inflammation. However, our understanding of their geographic distribution and climatic adaptation within China still needs to be improved. In this study, we screened the optimal predictive model (random forest model) to predict the potential suitable distribution of three *Sambucus* species (*Sambucus adnata*, *Sambucus javanica*, and *Sambucus williamsii*) across China under both current and future climate scenarios. Moreover, we identified key climate factors that influence their potential distributions. Our findings revealed that *S. adnata* and *S. javanica* are predominantly shaped by temperature seasonality and mean diurnal range, respectively, whereas *S. williamsii* is significantly affected by the precipitation of the wettest month. Currently, *S. williamsii* is primarily distributed in north and central south China (covering 9.57 × 10^5^ km^2^), *S. javanica* is prevalent in the south and east regions (covering 6.41×10^5^ km^2^), and *S. adnata* predominantly thrives in the southwest China (covering 1.99×10^5^ km^2^). Under future climate change scenarios, it is anticipated that *S. adnata* may migrate to higher latitudes while *S. javanica* may shift to lower latitudes. However, potentially suitable areas for *S. williamsii* may contract under certain scenarios for the years 2050 and 2090, with an expansion trend under the SSP585 scenario for the year 2090. Our study emphasizes the importance of climatic variables in influencing the potential geographic distribution of *Sambucus* species. These findings provide valuable theoretical insights for the preservation, cultivation, and utilization of *Sambucus* medicinal plant resources in the context of ongoing climate change.

## 1 Introduction

*Sambucus* genus plants hold significant medicinal value in China and have been extensively utilized for centuries owing to their multifaceted pharmacological properties (Wieland et al., 2021; Waswa et al., 2022). The initial classification of the *Sambucus* genus under the *Caprifoliaceae* family was later revised to *Adoxaceae*, incorporating genetic evidence and morphological comparisons, including nucleotide sequences and preliminary morphology. The genus *Sambucus* comprises approximately 20 species distributed in tropical and subtropical regions (Li et al., 2021). In China, the primary Sambucus species include *Sambucus adnata*, *Sambucus javanica*, and *Sambucus williamsii* (Liao et al., 2005; Xiao et al., 2016; Yuan et al., 2020). *S. adnata*, a perennial tall herb or subshrub, is commonly employed to treat fractures, traumatic injuries, and chronic nephritis diseases (Yuan et al., 2020; Li et al., 2021; Waswa et al., 2022). *S*. *javanica*, a perennial herb with multiple pharmacological functions, including anti-inflammatory and liver protection, is generally used to treat rheumatism, chronic inflammation of the airways, and viral hepatitis (Liao et al., 2005; Zhang et al., 2010; Chen et al., 2020). In addition, *S. williamsii*, another well-known herb, exhibits notable anti-inflammatory and fracture-healing properties and is frequently used to treat fractures and joint illness (Lv et al., 2015; Xiao et al., 2016). In recent years, herbal remedies for treating respiratory ailments have gained increased attention, particularly in the context of global respiratory infectious diseases such as COVID-19 (Wieland et al., 2021). Nevertheless, despite the recognized pharmacological worth of *Sambucus* plants (Waswa et al., 2022), there remains a lack of clarity regarding their geographical distribution and conservation status.

Climate change has been identified as the primary threat to biodiversity in the next century, with an estimated one-quarter of plant species at risk of extinction (Outlook, 2010; Dawson et al., 2011; Roman-Palacios and Wiens, 2020). According to the IPCC fifth assessment report, the global average temperature is projected to increase by 0.3 to 4.8°C by the end of this century (Change, 2013). Under such rapidly changing climate conditions, numerous terrestrial plants will modify their physiological, genetic, and community structure characteristics to shift their spatial distribution towards higher elevations and latitudes (Chen et al., 2011; Shen and Wang, 2013; Becklin et al., 2016; Yi et al., 2018). While *Sambucus* plants offer substantial economic and pharmacological value, their wild populations are limited, making it challenging to meet future demand, particularly in light of rapid climate change (Wang et al., 2021). Therefore, investigating the suitable geographical distribution of *Sambucus* species under climate change can serve the dual purpose of enhancing their commercial value and health benefits while preserving their natural habitat.

Global warming primarily results from the increased anthropogenic emissions of atmospheric greenhouse gases (Kim and Bae, 2021). To better understand the correlation between different socioeconomic development patterns and climate change risks, the Coupled Model Intercomparison Project Phase 6 (CMIP6) proposed four shared socioeconomic pathways (SSPs) based on radiative forcing levels and current national circumstances (O'Neill et al., 2014). Numerous studies have demonstrated that climate factors play a crucial role in shaping species distribution and can be used to model habitat changes in response to future climate scenarios (Kaky and Gilbert, 2017; Shrestha et al., 2018). These findings have significant implications for the conservation of medicinal plant resources, including *Coptis* (Li et al., 2020), *Artemisia annua L.* (Wang et al., 2022), *Homonoia riparia* (Yi et al., 2016), and *Scutellaria baicalensis* (Xu et al., 2020). In addition to climate factors, soil nutrients, and topographic conditions have long been recognized as key drivers of changes in species distribution that limit resources access to wild plant populations (Chytrý et al., 2003; Soberon and Peterson, 2005). Recent studies have shown that topographic conditions control the redistribution of solar radiation and precipitation (Cantón et al., 2004), while soil nutrients indicate the availability of belowground resources (John et al., 2007). Unfortunately, the critical limiting factors determining the potential distribution of *Sambucus* plants remain unclear.

In recent years, the rapid development of species distribution models (SDMs), based on the ecological and distributional characteristics of species, has provided scientific guidance for prioritizing species conservation (Zhang et al., 2018; Li et al., 2020; Rousseau and Betts, 2022; Zhao et al., 2023). Using the occurrence of target species and environment variables, SDMs can simulate the potential distribution of suitable habitats for species under climate change (Yi et al., 2016; Wang et al., 2022). However, it is important to note that the accuracy of SDMs relies on selecting appropriate species distribution models, which is critical for formulating effective conservation strategies. Over the past two decades, several noteworthy species distribution models have been formulated, including the generalized linear model (GLM, Lehmann et al., 2002), maximum entropy model ((MaxEnt, Phillips et al., 2006), random forest model (RF, Evans et al., 2011), genetic algorithms for rule set generation (GRAP, Stockwell and Peters, 1999), and ecological niche factor analysis (ENFA, Hirzel et al., 2002). The GLM model is frequently employed by scholars due to its simplicity and convenience, effectively handling the relatively simple relationship between response variables and environmental variables (Maria and Udo, 2017; Gábor et al., 2022). Similarly, the MaxEnt model, based on maximum entropy theory, requires only a small sample size to generate reliable results and has become one of the most popular species distribution models (Hernandez et al., 2006; Yi et al., 2016). Meanwhile, the RF model is known for being one of the best performing classification regression tree models, which can capture the complex nonlinear relationships among multiple ecological variables (Mi et al., 2017; Mohapatra et al., 2019; Rather et al., 2020). Although these species distribution models have been well evaluated in most studies, identifying the optimal model for accurately predicting the potential distribution of different plant species remains controversial.

This study focuses on investigating the implications of climate change on the potential distribution of three *Sambucus* species (*S. adnata*, *S. javanica*, and *S. williamsii*) in China. To achieve this, our study has three primary objectives: (1) to conduct a comparative analysis of the performance of three models (GLM, MaxEnt, and RF); (2) to simulate potential suitable distribution of the three species under current and future climate scenarios (SSP 2-4.5 and SSP 5-8.5) at two time points (2050 and 2090) in China; (3) to evaluate the relative importance of bioclimate, soil, and topographic factors in the spatial distributions of three *Sambucus* species. We hope this study can provide a basis for the scientific conservation and effective utilization of these important medicinal plants in China.

## 2 Materials and methods

### 2.1 Species occurrence data

**Figure 1:**
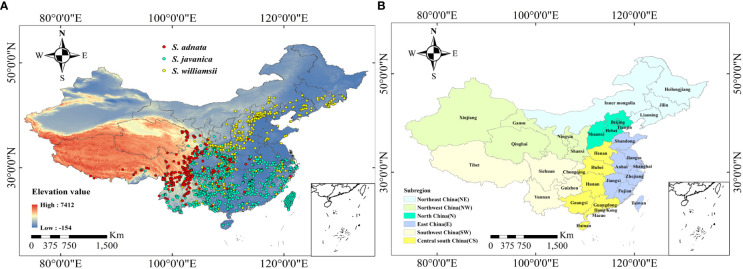
Original occurrence records of three *Sambucus* species in China. Occurrence records of *S. adnata*, *S. javanica* and *S. williamsii* in China **(A)**. Red points indicate *S. adnata*, blue points indicate *S. javanica* and golden points indicate *S. williamsii*. **(B)** The subregions division of China, including Northeast China (NE), Northwest China (NW), North China (N), East China (E), Southwest China (SW), and Central south China (CS).

We collected the specimen records of *S. adnata*, *S. javanica*, and *S. williamsii* dating back to the year 1955 through online databases, including the Global Biodiversity Information Facility (GBIF, https://doi.org/10.15468/dl.xyt3gh) and Chinese Virtual Herbarium (CVH, https://www.cvh.ac.cn/). To ensure the accuracy of geographical locations, we excluded problematic records with duplicate information (i.e., same sampling time and location), ambiguous geo-location and time, and artificially planted specimen information. For records lacking precise geographic coordinates, we used the Baidu Map Picking System to obtain the corresponding longitude and latitude. To minimize the spatial autocorrelation of species distribution points, we kept only one record closest to the centroid within each 5 km×5 km grid cell, using the “spThin” package in R (Aiello-Lammens et al., 2015). In the end, we obtained a total of 239 records for *S. adnata*, 863 records for *S. javanica*, and 509 records for *S. williamsii* in China (**Figure 1A**). Moreover, considering the spatial heterogeneity of administrative units and climatic conditions, we divided all provinces in China into six subregions: northeast, northwest, north, east, southwest, and central south (**Figure 1B**).

### 2.2 Selection and preprocessing of environmental variables

In this study, we initially selected 28 environmental factors (as detailed in **Table S1**) that may affect the spatial distribution of *S. adnata*, *S. javanica*, and *S. williamsii*. We categorized these factors into three distinct categories: bioclimatic, topographic, and soil factors. The bioclimatic dataset consisted of nineteen variables obtained from the WorldClim database, each with a spatial resolution of 5 km × 5 km (Hijmans et al., 2005). These variables encompassed essential climate parameters such as annual mean precipitation, annual mean temperature, minimum monthly precipitation, and maximum monthly precipitation, spanning the period from 1970 to 2000. This dataset effectively captured both annual and seasonal variations in temperature and precipitation, providing a comprehensive depiction of the prevailing climatic conditions. To project the gradual shifts in the potential distribution of *Sambucus* species in response to climate change, we utilized both current climate data and future climate projections for the 2050s and 2090s. The 2050s represent the mean values for the period 2041-2060, while the 2090s correspond to the average conditions projected for the years 2081-2100. This implies that the selected variables could effectively reflect the climatic conditions at the specified occurrence coordinates. Shared socioeconomic pathways are often used to model and represent the likely economic patterns of future societies and the resulting emissions of greenhouse gases and air pollutants. To evaluate the potential impact of climate change on these three *Sambucus* species, we incorporated future climate data estimated by eight global climate models: ACCESS-CM2, BCC-CSM2-MR, CMCC-ESM2, GISS-E2-1-G, IPSL-CM6A-LR, MIROC6, MPI-ESM1-2-HR and MRI-ESM2-0. Specifically, we integrated two shared socioeconomic pathways climate scenarios, SSP245 (moderate scenario) and SSP585 (pessimistic scenario), for the years 2050 and 2090 to accurately predict the potential distribution of these three species. For each future climate scenario, we calculated the mean values of 19 climate variables across the eight climate models.

In addition to bioclimatic factors, we recognized the importance of topographic and soil factors as key drivers of species distribution, as underscored in previous studies (Svenning et al., 2009; Buri et al., 2017). Specifically, we incorporated the latest digital elevation model (DEM) from the Shuttle Radar Topography Mission (SRTM) (Jarvis et al., 2008). Elevation, aspect, and slope were extracted from the SRTM with a spatial resolution of 90 m and subsequently resampled to a coarser resolution of 5 km to maintain consistency with other data layers. Furthermore, we considered six soil parameters crucial for plant growth, including soil pH and total nitrogen (**Table S1**). These soil variables were sourced from the SoilGrids database (Hengl et al., 2017) at a sampling depth from 5 to 15 cm with a spatial resolution of 1 km and were resampled to a spatial resolution of 5 km.

The selection of environmental variables has a significant impact on the accurate evaluation of species’ habitat suitability. High collinearity between environmental variables can reduce the accuracy of the species distribution models (SDMs) (Cruz-Cárdenas et al., 2014). Firstly, we performed Spearman correlation analysis using the ‘ggcorr’ function in the corrplot R package to exclude predictors with correlation coefficients greater than 0.8 (Pearson et al., 2007). Furthermore, we automatically selected a subset of predictors for each species by iteratively excluding variables with the variance inflation factor (VIF) greater than 10 using the ‘vifstep’ function of the usdm R package (Naimi et al., 2014). In the end, we retained 10 environmental variables for modeling *S. adnata* (**Figure S1A**), 12 for modeling *S. javanica* (**Figure S1B**), and 11 for modeling *S. williamsii* (**Figure S1C**).

### 2.3 Model implementation and accuracy assessment

To identify the most accurate species distribution models for *S. adnata*, *S. javanica*, and *S. williamsii* in China, we evaluated three widely used SDMs: generalized linear models (GLM; Nelder and Wedderburn, 1972), maximum entropy models (MaxEnt; Phillips et al., 2006), and random forest models (RF; Breiman, 2001). In addition to the existing presence points of the three species, we randomly generated 5000 background points for each species in China. Then, we randomly divided the dataset into a 30% test set and a 70% training set with 10 repetitions using bootstrap validation to reduce the uncertainty. The sdm package in R 4.2.1 was used to simulate the three SDMs models (Naimi and Araujo, 2016).

The accuracy of the three species distribution models was assessed mainly by four complementary metrics, including the area under the receiver operating characteristic (ROC) curve (AUC), true skill statistics (TSS), Sensitivity and Specificity (Allouche et al., 2006; Lobo et al., 2008; Liu et al., 2010). The AUC is a threshold-independent evaluation criterion (Hanley and McNeil, 1982), which is used to assess the ability of the predictive model (Phillips, 2008). In this study, three levels were used for model evaluation, as follows: AUC values below 0.7 were considered poor, values between 0.7 and 0.9 were considered moderate, and values greater than 0.9 were considered excellent (Li et al., 2020). In addition, we used another model evaluation metric, the TSS, which is calculated as the true positive rate minus the false positive rate (Allouche et al., 2006). TSS values between 0.4 and 0.6 were categorized as “fair,” while those between 0.6 and 1 were categorized as “good” (Xiao et al., 2019). Sensitivity is the probability that correctly predicts a presence, while Specificity is the probability that correctly predicts an absence (Liu et al., 2010). It is worth noting that the TSS value equals to Sensitivity value plus Specificity minus 1.

Finally, the model with the highest AUC, TSS, Sensitivity, and Specificity values was selected as the best predictive model. To further elucidate the effects of environmental factors on species distribution, we calculated the relative contribution of each predictor variable to the distribution model. Subsequently, response curves were generated to investigate the intrinsic relationships between the key factors affecting the potential distribution of the three species.

### 2.4 Evaluation of the dynamics of species habitat suitability

We used the best model to predict the spatial distribution of *S. adnata*, *S. javanica*, and *S. williamsii* under current and multiple future climate scenarios (SSP245-2050, SSP245-2090, SSP585-2050, and SSP585-2090). Using the species distribution model outputs, we transformed the continuous suitability scores (ranging from 0-1) into habitat suitability maps in ArcMap 10.8. To assess changes in the predicted distribution of each species, we constructed binary distribution maps for both current and future climate scenarios using the SDMtoolbox. The areas of suitable and unsuitable zones of three species were calculated. Firstly, we employed the sdm package in R to calculate the minimum suitability score threshold of each species. Below the score threshold represents unsuitable for the survival of the species (Absence), and greater than or equal to the threshold represents suitable for the survival (Presence). Secondly, we calculated the suitable and unsuitable survival areas in the SDMtoolbox of ArcGIS. Specifically, we quantified trends in future species suitability areas by calculating species distribution under future climate scenarios relative to current distributions. To evaluate changes in suitability, we classified the results into three categories: contraction trend, no change trend, and expansion trend.

## 3 Results

### 3.1 Model assessment

All three species (*S. adnata*, *S. javanica*, and *S. williamsii*) performed well under current climate conditions in all species distribution models (GLM, MaxEnt, and RF; AUC > 0.9, TSS > 0.6), except for the *S. williamsii* in the GLM model (AUC = 0.84, TSS = 0.56) (**Table 1**). The RF models had the best predictive performance for all three species, with AUC ranging from 0.96 to 0.98 and TSS ranging from 0.8 to 0.86, while the GLM models had lower suitability, with AUC ranging from 0.84 to 0.92 and TSS ranging from 0.56 to 0.75 (**Table 1**). In addition, the RF models had the highest Specificity and Sensitivity values, while the GLM models had the lowest Specificity and Sensitivity values. Therefore, we used the RF models to predict the current and potential future distribution of all three species.

**Table 1:** Accuracy evaluation of GLM, MaxEnt and RF models in predicting the potential distribution of *S. adnata*, *S. javanica* and *S. williamsii* in China.

species	methods	AUC	TSS	Sensitivity	Specificity
*S. adnata*	GLM	0.92	0.71	0.91	0.79
	MaxEnt	0.96	0.81	0.93	0.88
	RF	0.98	0.86	0.94	0.92
*S. javanica*	GLM	0.91	0.75	0.98	0.74
	MaxEnt	0.93	0.77	0.98	0.79
	RF	0.97	0.84	0.94	0.90
*S. williamsii*	GLM	0.84	0.56	0.89	0.66
	MaxEnt	0.91	0.7	0.92	0.78
	RF	0.96	0.8	0.89	0.91

AUC, TSS, Sensitivity, and Specificity are four indicators for model accuracy evaluation, and the larger their values are, the higher the model accuracy is. GLM, generalized linear model; MaxEnt, maximum entropy model; RF, random forest model.

### 3.2 Influence of predictor variables

**Figure 2:**
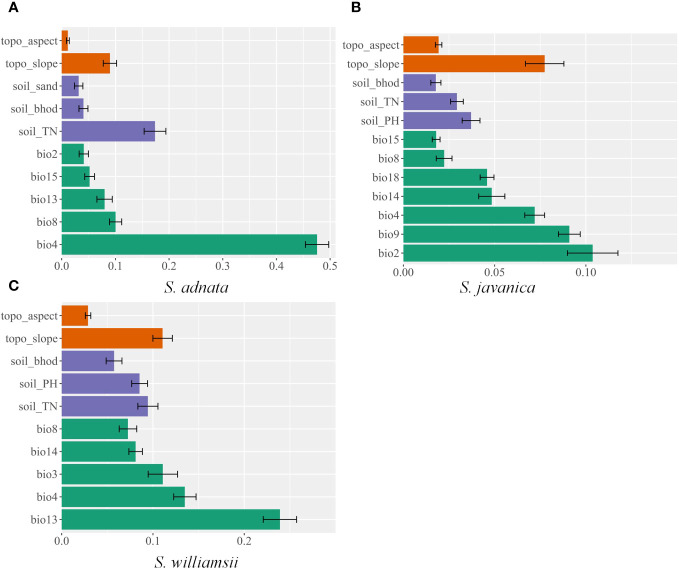
The relative contribution of environmental variables used in this study in predicting the current distribution of *S. adnata* **(A)**, *S. javanica***(B)**, and *S. williamsii* **(C)** in China.

**Figure 3:**
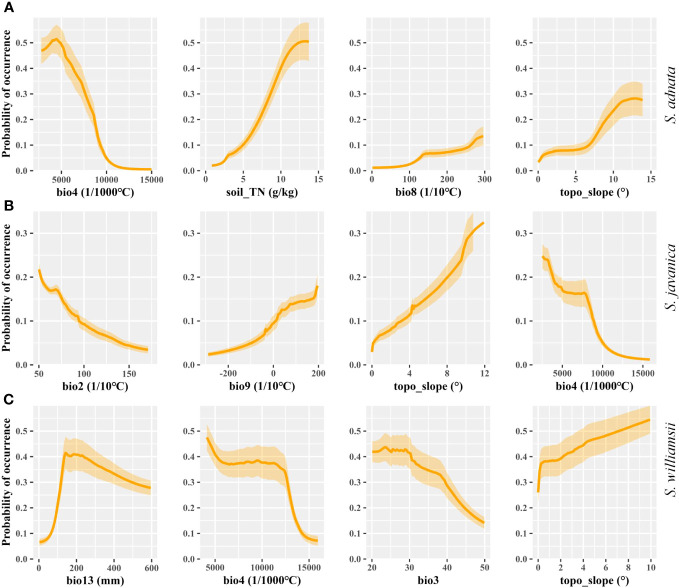
The response curves for the four most important contributing environment variables for *S. adnata* **(A)**, *S. javanica* **(B)**, and *S. williamsii* **(C)** under the current climate conditions in China, which reflect the relationship between environmental variables and probability of occurrence of species.

Overall, the geographic distribution of *S. adnata*, *S. javanica*, and *S. williamsii* was primarily influenced by climatic factors, followed by soil and topography factors (**Figure 2**). Specifically, for *S. adnata*, the four most important predictor variables were temperature seasonality (bio4, 47.6%), soil total nitrogen (soil_TN, 17.4%), mean temperature of the wettest quarter (bio8, 10%), and slope (topo_slope, 9%) (**Figure 2A**). To understand how each key environmental variable influenced potential distribution of all three species, we plotted variable response curves (**Figure 3**). The probability of occurrence of *S. adnata* was negatively correlated with bio4, with an exception of a slight initial positive correlation (**Figure 3A**). In contrast, within a specific range, the probability of occurrence of *S. adnata* was positively correlated with the soil_TN, topo_slope, and bio8 (**Figure 3A**).

For *S. javanica*, the most important factors for potential distribution were the mean diurnal range (bio2, 10.4%), mean temperature of the driest quarter (bio9, 9.1%), slope (topo_slope, 7.7%), and temperature seasonality (bio4, 7.2%) (**Figure 3B**). There was a positive relationship between the occurrence probability for *S. javanica* and bio9 and topo_slope, while a negative relationship between bio2 and bio4 (**Figure 3B**).

For *S. williamsii*, the four most dominant contributing environmental variables were the precipitation of wettest month (bio13, 23.9%), temperature seasonality (bio4, 13.5%), isothermality (bio3, 11.1%), and slope (topo_slope, 11.1%). Moreover, we found that there was a unimodal relationship bio13 and the probability of occurrence of *S. williamsii* (**Figure 3C**). Meanwhile, the probability of occurrence of *S. williamsii* was positively associated with the topo_slope, but negatively correlated with bio3 and bio4 (**Figure 3C**).

### 3.3 Predicting distribution under current and future conditions

**Figure 4:**
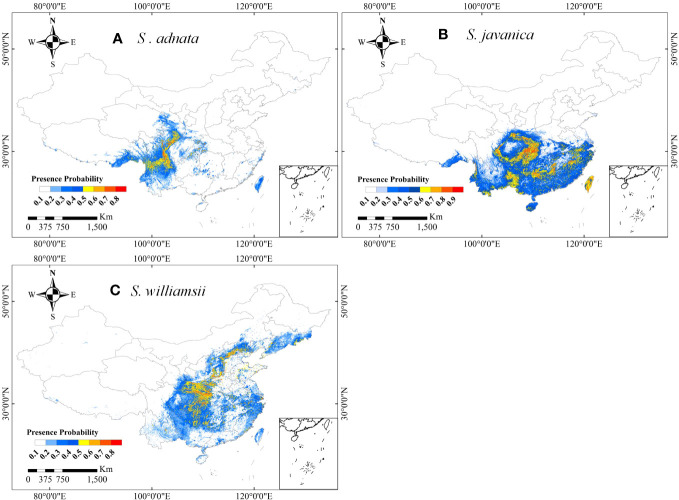
The potential distributions of *S. adnata* **(A)**, *S. javanica* **(B)**, and *S. williamsii* **(C)** under current climate conditions in China. The legend colors indicate the differences of presence probability of species in different area.

The distribution patterns of *S. adnata*, *S. javanica*, and *S. williamsii* varied widely across China under current climatic conditions (**Figure 4**). Overall, all three species have high habitat suitability scores, with values greater than or equal to 0.8. Specifically, *S. adnata* was mainly distributed in northwest and southwest China, with only small areas in central south China (**Figures 1B**, **4A**). The highly suitable areas for *S. adnata* were located throughout the southwest China, extending north to the southern part of northwest China (**Figures 1B**, **4A**). Moreover, *S. javanica* had suitable areas distributed widely in almost all southern regions of China. Notably, the highly suitable areas for *S. javanica* occurred at the boundaries of three subregions: the northwest and southwest edges of China, the southwest and central south edges of China, and the entire Taiwan Province (**Figures 1B**, **4B**). In addition, *S. williamsii* was mainly located in north, east, southwest and central south China, with only a small portion in northwest China (**Figures 1B**, **4C**). The highly suitable areas were in the border of southwest and central south China, and a small southern region of northwest China.

**Figure 5:**
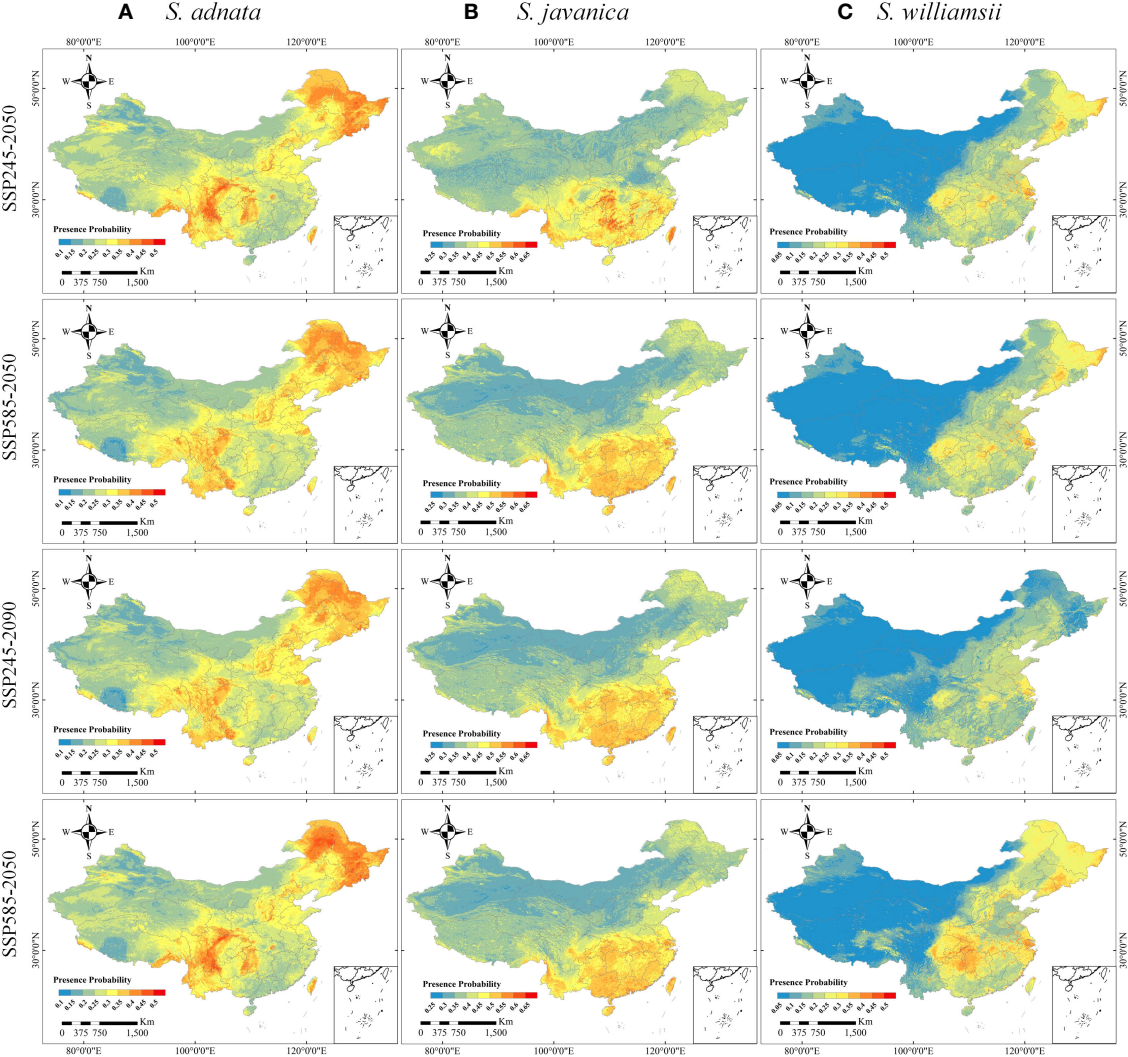
The potential distributions of *S. adnata* **(A)**, *S. javanica* **(B)**, and *S. williamsii* **(C)** under SSP245-2050, SSP585-2050, SSP245-2090 and SSP585-2090 scenarios in China. The legend colors indicate the differences of the presence probability of species in a different area.

The potential distributions of the three species under the four future climate scenarios (SSP245-2050, SSP245-2090, SSP585-2050, and SSP585-2090) were highly divergent compared to their current distributions (**Figure 5**). Specifically, the potential suitable distribution of *S. adnata* was expected to largely expand under future climate scenarios, with significant increases in relatively high suitability areas in northeast and north China (**Figures 1B**, **5A**). Similarly, except for northwest China, the future potential suitability of *S. javanica* would be expanded in other regions of China (**Figures 1B**, **5B**). However, the highly potentially suitable areas of *S. javanica* would basically be maintained in the current distribution areas (**Figure 5B**). Furthermore, the potential future suitability of *S. williamsii* would be expanded in northeast, north, and east China. Notably, the potentially suitable areas of *S. williamsii* increased most significantly under the SSP585-2090 scenario, especially in northeast China (**Figures 1B**, **5C**).

**Figure 6:**
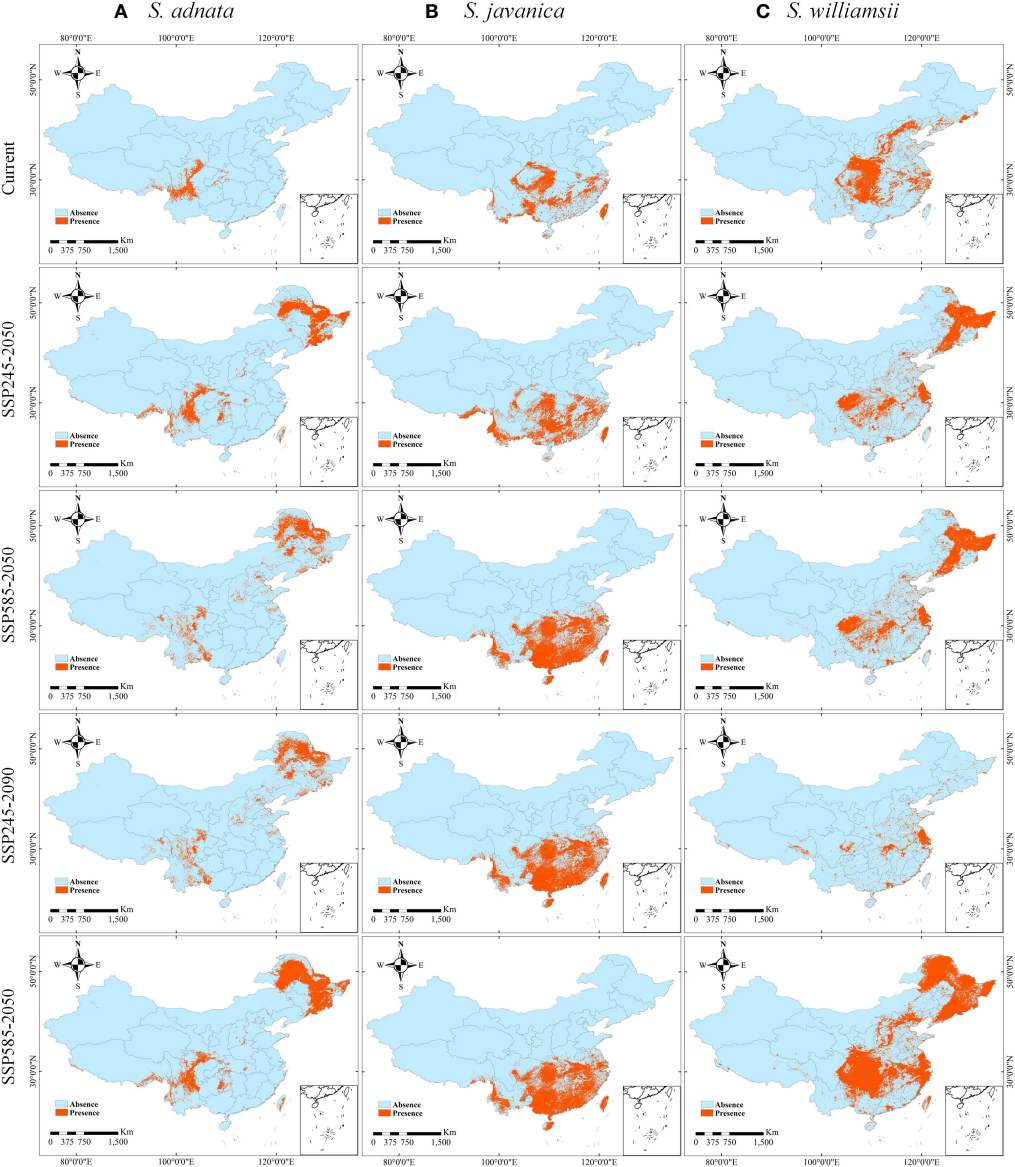
The presence and absence distributions of *S. adnata* **(A)**, *S. javanica* **(B)**, and *S. williamsii* **(C)** under current and future climatic conditions in China. The future climate conditions including SSP245-2050, SSP585-2050, SSP245-2090 and SSP585-2090 scenarios.

To further quantify the areas of suitable regions, we mapped the binary distribution of the three species under current and future climate scenarios (SSP245-2050, SSP245-2090, SSP585-2050, and SSP585-2090) (**Figure 6**). The areas of suitable zones for *S. adnata* under current and four future climate scenarios were calculated: 1.99×10^5^, 7.14×10^5^, 5.59×10^5^, 5.59×10^5^, and 8.75×10^5^ km^2^, respectively (**Figure 6A**, **Table S2**). Similarly, the areas of suitable zones for *S. javanica* under current and future climate scenarios were 6.41×10^5^, 9.46×10^5^, 13.54×10^5^, 13.54×10^5^, and 13.54×10^5^ km^2^, respectively (**Figure 6B**, **Table S2**). Finally, the areas of suitable zones for *S. williamsii* under current and future climate scenarios were 9.57×10^5^, 10.79×10^5^, 10.79×10^5^, 3.55×10^5^, 21.68×10^5^ km^2^, respectively (**Figure 6C**, **Table S2**).

### 3.4 Future changes in suitable habitats

**Figure 7:**
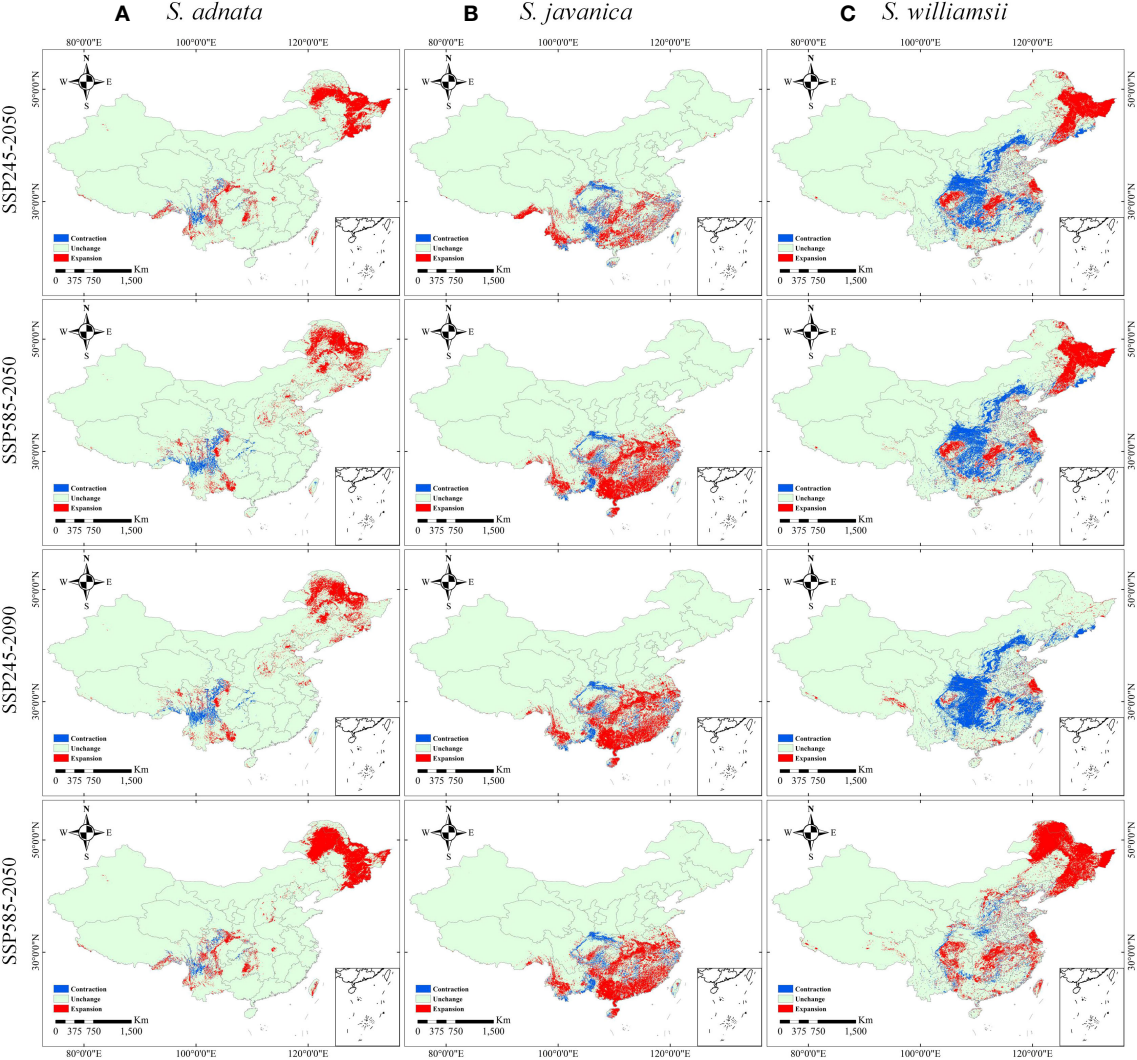
Changes in the distribution of *S. adnata* **(A)**, *S. javanica* **(B)**, and *S. williamsii* **(C)** under SSP245-2050, SSP585-2050, SSP245-2090, and SSP585-2090 scenarios compared to current distribution in China. The legend colors indicate as follows: indicolite green denotes unchanged areas, red indicates expansion areas, and blue represents contraction areas.

Our analysis of four future climate scenarios revealed significant differences in the expansion and contraction trends of all three species (**Figure 7**, **Table S3**). The expansion of *S. adnata* was observed in northeast China, while its contraction occurred in current high suitable zone (**Figure 7A**). Similarly, *S. javanica’s* expansion was seen in the southern part of China, while its contraction occurred in the current high suitability area. (**Figure 7B**). In addition, the potentially suitable distribution areas of *S. williamsii* varied significantly under different future climate scenarios (**Figure 7C**). We observed a contraction trend in north and parts of northwest China, and the borders of southwest and central south China under SSP245-2050, SSP245-2090, and SSP585-2050 scenarios. Conversely, northeast China showed a large expansion trend under the SSP585-2090 scenario.

## 4 Discussion

The genus *Sambucus* has significant roles in traditional Chinese medicine due to its pharmacological properties, such as the treatment of respiratory illness (Wieland et al., 2021), bone fractures (Liao et al., 2005; Lin et al., 2009; Wang et al., 2020) and diabetes (Waswa et al., 2022). In this study, using the best predictive model (RF model), we mapped, for the first time, the potential suitable distribution of three *Sambucus* species, *S. adnata*, *S. javanica*, and *S. williamsii*, under the current and future climate scenarios in China. Moreover, we further explored potential drivers influencing the habitat distribution of these three *Sambucus* species.

### 4.1 Model predictive performance

According to the evaluation of AUC, TSS, Specificity, and Sensitivity values, our results indicated that the GLM model was less predictive than the MaxEnt and RF models for the three *Sambucus* species (**Table 1**). This suggests that GLM models are more appropriate for analyzing linear correlations, but cannot adequately capture nonlinear relationships between high-dimensional variables (Elith and Graham, 2009; Mainali et al., 2015). In contrast, MaxEnt models offer several advantages for species distribution prediction, such as an easily interpreted modeling mechanism (Zhang and Wang, 2022) and not requiring background data (Elith et al., 2011). However, it is worth noting that the parameter settings of the MaxEnt model need to be optimized for accurate prediction results (Radosavljevic and Anderson, 2014; Morales et al., 2017; Lissovsky and Dudov, 2021). Compared to GLM and MaxEnt models, our findings indicated that that RF performed the best in predicting the potentially suitable distribution of *Sambucus*, which is consistent with previous studies (González-Irusta et al., 2015; Mi et al., 2017; Čengić et al., 2020). Notably, RF models offer the benefits of computational efficiency and reliable prediction results without parameter adjustment, which are applicable to both large and small samples (Luan et al., 2020; Zhang et al., 2020; Zhao et al., 2022). Overall, our study emphasizes the importance of selecting appropriate modeling techniques for predicting the potential distribution of species.

### 4.2 Habitat distribution and key environmental factors under current environment

Environmental heterogeneity can influence distribution and abundance of species by altering conditions in temperature, precipitation, light, and nutrient availability (Palmer and Dixon, 1990; Wang et al., 2012; Ehrlén and Morris, 2015). Our findings indicated that climate drivers were more influential in determining the habitat distribution of *S. adnata*, *S. javanica*, and *S. williamsii* than topographic and soil drivers, which is consistent with recent studies (Dyderski et al., 2018; Yue et al., 2019; Naudiyal et al., 2021; Wang et al., 2022).

Specifically, we observed a strong negative effect of the temperature seasonality range (bio4) on the geographical distribution of all three species, particularly *S. adnata* (**Figure 3**). The observed distribution of *Sambucus* species in central, and southwestern China underscores their preference for stable temperature habitats (**Figure 4**). Furthermore, *Sambucus* plants exhibit a propensity for thriving within forested environments, benefiting from the temperature-buffering effect the forest canopy offers (De Lombaerde et al., 2022). Moreover, our findings revealed that the mean temperature of the wettest quarter (bio8) and the mean temperature of the driest quarter (bio9) played pivotal roles in shaping the distribution of *S. adnata* and *S. javanica*, respectively. This finding aligns with previous studies, which have consistently highlighted the positive influence of temperature on plant growth by extending the growing season and accelerating the phenological process (Zhang et al., 2015; Ma and Sun, 2018; Zhang et al., 2018). In addition, recent studies have also emphasized the importance of precipitation as a major factor influencing the distribution and survival of plant species (Petrie et al., 2016; Wang et al., 2018). In contrast to the responses of *S. adnata* and *S. javanica* to temperature, *S. williamsii* was more sensitive to variations in precipitation than temperature (**Figure 3**). Similar to a recent study (Naudiyal et al., 2021), the precipitation of wettest month (bio13) was identified as the most influential variable in determining the stable habitat distribution of *S. williamsii*, spanning a range from 0 to 270 mm. This underscores the notion that moderate precipitation levels confer benefits to plant growth and seedling survival (Petrie et al., 2016), while excessive soil water can create anaerobic conditions, detrimentally impacting plant metabolism and constraining growth (Zhang et al., 2019). Collectively, these findings underscore the pivotal role of temperature and precipitation as prerequisites for suitable distributions of *Sambucus* species, concurrently highlighting variations in ecological suitability among the three species.

In addition to climatic factors, topographic variables, especially slope, emerged as pivotal determinants shaping the habitat distributions of *S. adnata*, *S. javanica* and *S. williamsii* in the central and southwest China (**Figure 3**), where the genus *Sambucus* was mainly distributed (**Figure 4**). This aligns with previous research that underscores the provision of ample light to understory vegetation on steep slopes, facilitating their growth and regeneration (Austin and Van Niel, 2011; Chen et al., 2018). Additionally, our findings highlighted the varying impact of soil organic nitrogen (soil_TN) on the distribution patterns of the three species. Notably, *S. adnata* displayed a stronger positive correlation with soil_TN than *S. javanica* and *S. williamsii*, supporting prior studies that have elucidated the differential influence of soil nutrient availability on species distributions (Swaine, 1996; Lévesque et al., 2016; Walthert and Meier, 2017). Therefore, regions characterized by high soil nutrient availability are more suitable for the distribution of *S. adnata*.

It is worth noting that global climate change profoundly impacts soil properties across various aspects (Certini and Scalenghe, 2023). For instance, recent studies have demonstrated effects on soil pH patterns across different depths and grassland types, as well as on soil nutrient cycling processes (Gao et al., 2014; Sun et al., 2021b). Moreover, the regulation of moisture and temperature by climate change has a direct impact on soil biogeochemical responses, which in turn affect plant growth and distribution (Classen et al., 2015; Certini and Scalenghe, 2023).

### 4.3 Future distribution of *Sambucus* under climate change

Several recent studies have provided evidence indicating that climate change would significantly alter species ranges (Chen et al., 2011; Scheffers et al., 2016; Li et al., 2019), resulting in species migrating northward or to higher latitudes (Hickling et al., 2006; Bertrand et al., 2011). To simulate the maximum potential impact of greenhouse gas emissions on the *Sambucus* species, their potential distributions were predicted by our most robust prediction model (i.e., RF) across multiple future climate scenarios. Our findings indicate that the potentially suitable habitats of *S. adnata*, *S. javanica*, and *S. williamsii* are differ significantly under the impacts of climate change.

The potential suitable areas of *S. adnata* and *S. javanica* were predicted to expand significantly under future climate scenarios, but the directions of expansion were different. Specifically, *S. adnata* would shift its potential habitats to higher latitudes, while *S. javanica* would shift to lower latitudes. Contrary to previous studies that found negative effects of the habitat degeneration or population loss due to climate warming (Brecka et al., 2018; Dyderski et al., 2018; Sun et al., 2021a), both *S. adnata* and *S. javanica* are expected to benefit from global warming, possibly due to their broad thermal tolerance ranges (Calosi et al., 2008). Studies have shown that species with narrow thermal tolerances are more vulnerable to climate change than those with broader thermal ranges (Jiguet et al., 2006; Khaliq et al., 2014).

With global warming occurs, animals and plants have been observed to shift their distribution areas to higher latitudes or elevations to adapt to the changing climate (Parmesan, 2006; Chen et al., 2011; Feeley, 2012; Lenoir and Svenning, 2013). However, the direction and magnitude of changes in the geographic distribution of different species vary considerably (Lenoir et al., 2008; Fei et al., 2017). The distributions of species are not regulated by temperature alone (Wisz et al., 2013; Grytnes et al., 2014), but are driven by a combination of ecological factors, particularly the combined effects of temperature and moisture conditions (Zu et al., 2021). Recent studies revealed that the distinct roles played by temperature and precipitation in shaping the geographic distribution of species determine the direction of species shift under climate change (Zu et al., 2021; Chen et al., 2022).

However, the potential suitable areas of *S. williamsii* are expected to exhibit significantly different contraction and expansion trends under various future climate scenarios. There will be a noticeable trend of contraction in parts of northwest China, and the borders of southwest and central south China under SSP245-2050, SSP585-2050, and SSP245-2090 scenarios, but a trend of expansion in northeast China under the SSP585-2090 scenario. Compared to the SSP245 scenario, future climate change under the SSP585 scenario will become increasingly complicated over time (Liu et al., 2022). By the end of this century, the potential suitable habitats of *S. williamsii* are predicted to contract under the SSP245 scenario, but to expand under the SSP585 scenario. This illustrates the dual impact of climate change on suitable habitats of organisms (Liu et al., 2022). However, the complexity of climate change also contributes to uncertainty in species distribution projections (Millar et al., 2007; Yousefpour et al., 2012; Lindner et al., 2014).

### 4.4 Prospect

Our study provides a framework to predict the current and future geographic distribution of plant species based on climate, soil, and topography factors. However, there are still some uncertainties in our findings. First, the accuracy of the model is critical to the prediction results and can be improved through parameter adjustments (Radosavljevic and Anderson, 2014). Second, while climate, soil, and topography factors play key roles in species distribution, other factors such as human activities, land use change (Yu et al., 2019), biological interaction (Boulangeat et al., 2012), dispersal distances and limitations (Peterson et al., 2008), also impact species distribution patterns. Due to the inherent challenges in quantifying certain aspects and the potential issue of multicollinearity arising from incorporating all variables in the model, our study primarily focused on examining the effects of key variables. Therefore, our results may exhibit slight variations from the actual distribution pattern of the three species, and subsequent studies can supplement our findings.

Notably, the impact of human activities on plant species distribution cannot be overlooked, as they have become increasingly influential in shaping suitable habitats for animals and plants (Xu et al., 2019). Specifically, the impact of human activities on the diversity and distribution of plant communities can be either positive or negative (Wang et al., 2022; Huang and Fu, 2023). Moreover, the structure and function of different plant communities show considerable variation in response to human activities (Sun et al., 2021b; Zha et al., 2022). Therefore, it is crucial to consider the effects of human activities on species distributions in future studies.

Despite these uncertainties, we have confidence in the predictions of the RF model, which provides scientific theoretical foundations for the conservation and exploitation of the three medicinal plants from *Sambucus*. Therefore, we suggest that the contraction areas of *S. adnata* (southwest China), *S. javanica* (southwest China), and *S. williamsii* (north, central south, and southwest China) should be prioritized for germplasm resource collection. The expansion areas of *S. adnata* (northeast China), *S. javanica* (east and central south China), and *S. williamsii* (northeast China) may be suitable for introduction and cultivation.

## 5 Conclusions

This study demonstrated the significant ecological habitat differences among *S. adnata*, *S. javanica*, and *S. williamsii* in China. Furthermore, our findings underscore the pivotal roles of temperature and precipitation in shaping the potentially suitable habitats for these species. Specifically, *S. adnata* displayed heightened sensitivity to temperature seasonality, while *S. javanica* exhibited a strong dependence on mean diurnal range, and *S. williamsii* demonstrated a pronounced reliance on precipitation of the wettest month. Currently, *S. williamsii* primarily occupies north and central south China (9.57 × 10^5^ km^2^), *S. javanica* is prevalent in the south and east regions (6.41×10^5^ km^2^), and *S. adnata* predominantly occupies southwest areas (1.99×10^5^ km^2^). Under future climate scenarios, we anticipated that *S. adnata* may shift to higher latitudes, while *S. javanica* may shift to lower latitudes. Our findings provide valuable insights into the potential impacts of climate change on the distributions of three *Sambucus* species in China. These insights can inform the development of effective conservation strategies, ensuring the preservation and sustainable management of these valuable medicinal plant resources.

## Data availability statement

The original contributions presented in the study are included in the article/**Supplementary Material**. Further inquiries can be directed to the corresponding author.

## Author contributions

WL: Conceptualization, Methodology, Formal analysis, Writing – review & editing. SH: Investigation, Formal analysis, Data curation, Writing – original draft. TY: Investigation, Writing – review & editing. PW: Methodology, Writing – review & editing. YM: Investigation, Writing – review & editing. MW: Investigation, Writing – review & editing. JL: Supervision, Writing – review & editing. ZL: Supervision, Writing – review & editing. JT: Conceptualization, Validation, Funding acquisition, Writing – review & editing.
