# Supplementary material for: Assessing the suitability and dynamics of three medicinal Sambucus species in China under current and future climate scenarios
Source: Front Plant Sci. 2023 Oct 19;14:1194444. doi: 10.3389/fpls.2023.1194444 (PMC10620941; doi:10.3389/fpls.2023.1194444)

**Assessing the suitability and dynamics of three medicinal *Sambucus* species in China under current and future climate scenarios**

**Weixue Luo ^1, 2†^, Shunxin Han^1†^, Ting Yu^1^, Peng Wang^1^, Yuxuan Ma^1^, Maji Wan^1^, Jinchun Liu^1, 2^, Zongfeng Li^1, 2^, Jianping Tao^1, 2*^**

^1^ Key Laboratory of Eco-environments in Three Gorges Reservoir Region (Ministry of Education), Chongqing Key Laboratory of Plant Ecology and Resources Research in Three Gorges Reservoir Region, School of Life Sciences, Southwest University, Chongqing, China

^2^ Chongqing Jinfo Mountain Karst Ecosystem National Observation and Research Station, Southwest University, Chongqing, China

**†Shared first authorship**

Email: W. Luo: luowx0305@swu.edu.cn; S. Han: hsx_1874@163.com;

T. Yu: 18290561848@163.com; P. Wang: wangp9909@126.com;

Y. Ma: myx19991001@163.com; M. Wan: wanmaji_361@163.com;

J. Liu: jinchun@swu.edu.cn; Z. Li: lizfswu@swu.edu.cn

***Corresponding author:** J. Tao: taojp@swu.edu.cn

Present address: Southwest University, No.2 Tiansheng Road, Beibei District, Chongqing 400715, China

**This file includes:**

Number of pages: 5; Number of tables: 3; Number of figures: 1

# Supplementary Figures and Tables

## Supplementary Tables

**Supplementary Table1** List of environmental variables considered in this study.

| Abbreviation | Description | Unit |
| --- | --- | --- |
| bio1 | annual mean temperature | 1/10℃ |
| bio2 | mean diurnal range (mean of monthly (max temp – min temp)) | 1/10℃ |
| bio3 | isothermality (bio2/bio7) (×100) | Unitless |
| bio4 | temperature seasonality (standard deviation×100) | 1/1000℃ |
| bio5 | max temperature of warmest month | 1/10℃ |
| bio6 | min temperature of coldest month | 1/10℃ |
| bio7 | temperature annual range | 1/10℃ |
| bio8 | mean temperature of wettest quarter | 1/10℃ |
| bio9 | mean temperature of driest quarter | 1/10℃ |
| bio10 | mean temperature of warmest quarter | 1/10℃ |
| bio11 | mean temperature of coldest quarter | 1/10℃ |
| bio12 | annual precipitation | mm |
| bio13 | precipitation of wettest month | mm |
| bio14 | precipitation of driest month | mm |
| bio15 | Precipitation of seasonality, coefficient of variation | Unitless |
| bio16 | precipitation of wettest quarter | mm |
| bio17 | precipitation of driest quarter | mm |
| bio18 | precipitation of warmest quarter | mm |
| bio19 | precipitation of coldest quarter | mm |
| soil_bhod | bulk density of the fine earth fraction | kg dm-3 |
| soil_ocd | organic carbon density | kg m-3 |
| soil_PH | PH （H_2_O） | Unitless |
| soil_sand | sand (>0.05 mm) in fine earth | % |
| soil_soc | soil organic carbon in fine earth | g kg-1 |
| soil_TN | soil total nitrogen | g kg-1 |
| topo_aspect | aspect | Unitless |
| topo_elev | elevation | m |
| topo_slope | slope | ° |

**Supplementary Table2** The area of presence and absence distribution of *S. adnata*, *S.javanica* and *S.williamsii* under current and future climate scenarios. Note: the unit of area in the table is 10,000 square kilometers (10^4^ km^2^).

| species | binary | Current | 2050 | | 2090 | |
| --- | --- | --- | --- | --- | --- | --- |
|  |  |  | SSP245 | SSP585 | SSP245 | SSP585 |
| *S. adnata* | absent | 939.94 | 889.08 | 904.61 | 904.61 | 872.97 |
|  | present | 19.88 | 71.39 | 55.86 | 55.86 | 87.49 |
| *S. jvanica* | absent | 895.74 | 865.85 | 825.04 | 825.04 | 825.04 |
|  | present | 64.09 | 94.61 | 135.43 | 135.43 | 135.43 |
| *S. williamsii* | absent | 864.12 | 852.58 | 852.58 | 924.96 | 743.63 |
|  | present | 95.70 | 107.88 | 107.88 | 35.50 | 216.83 |

**Supplementary Table3** The change area of *S. adnata*, *S. javanica* *and S. williamsii* under SSP245-2050, SSP585-2050, SSP245-2090 and SSP585-2090 scenarios compared to current distribution in China. Note: the unit of area in the table is 10,000 square kilometers (10^4^ km^2^).

| species | change | 2050 | | 2090 | |
| --- | --- | --- | --- | --- | --- |
|  |  | SSP245 | SSP585 | SSP245 | SSP585 |
| *S. adnata* | Contraction | 7.98 | 15.42 | 15.42 | 7.59 |
|  | Unchange | 893.16 | 893.69 | 893.69 | 877.76 |
|  | Expansion | 59.10 | 51.13 | 51.13 | 74.89 |
| *S. jvanica* | Contraction | 24.53 | 20.29 | 20.29 | 20.29 |
|  | Unchange | 881.42 | 849.67 | 849.67 | 8449.67 |
|  | Expansion | 54.28 | 90.28 | 90.28 | 90.28 |
| *S. williamsii* | Contraction | 77.43 | 77.43 | 97.25 | 21.74 |
|  | Unchange | 807.03 | 807.03 | 839.39 | 809.27 |
|  | Expansion | 75.77 | 75.77 | 23.60 | 129.23 |

## 1.2 Supplementary Figures

**Supplementary Figure1** Pearson correlation matrix of employed environmental variables for *S. adnate*(A)*, S. javanica*(B) *and S. williamsii*(C)*.* The correlation coefficient “r” takes values between -1 ~ 1, with r = -1 ~ 0 indicating negative correlation and r = 0 ~ 1 indicating positive correlation. If | r | ≥ 0.8, it means that the correlation is too high and the environment variable should be excluded.


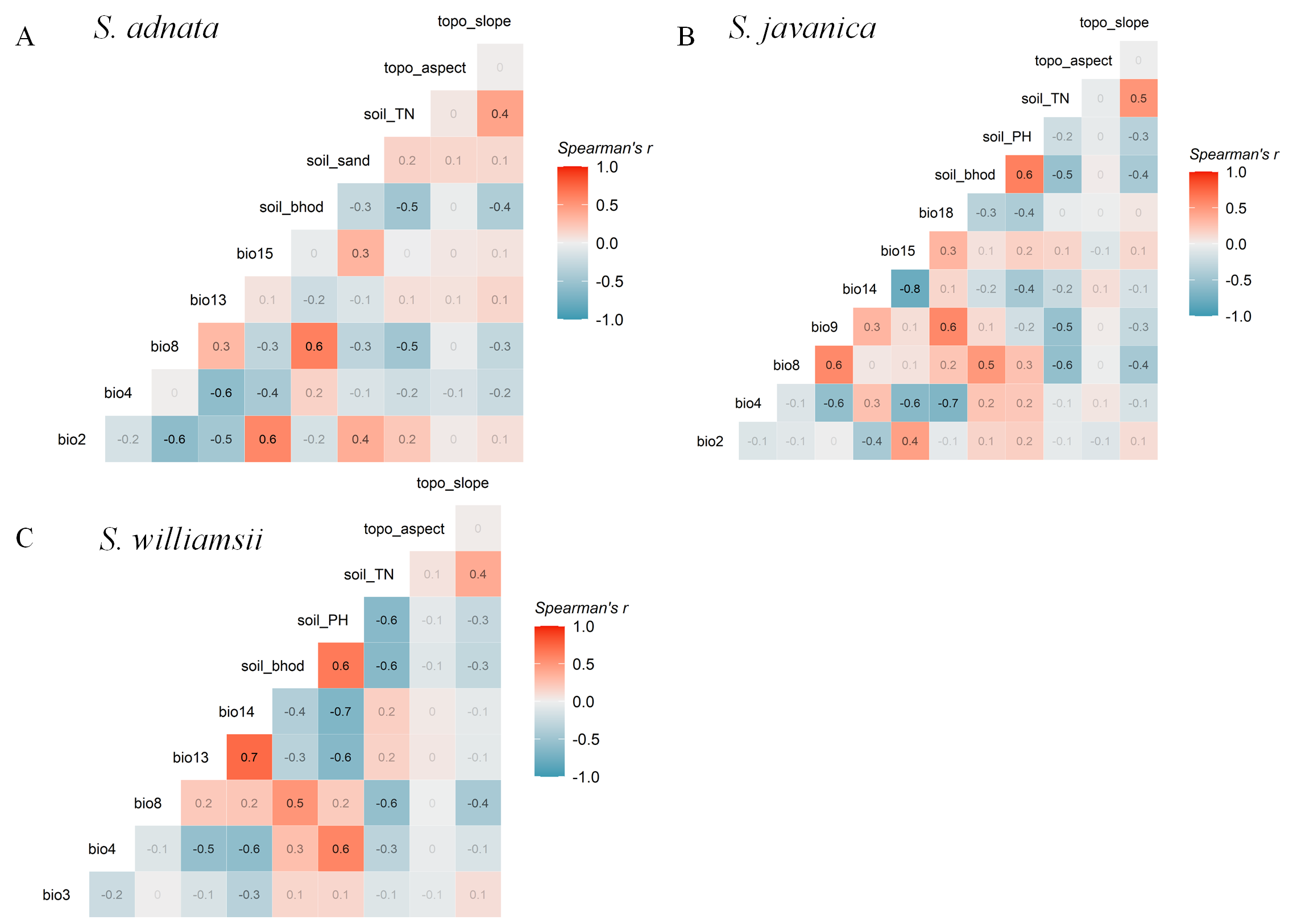

Supplement: Supplementary file 1 [file DataSheet_1.docx]
